# *In vitro* selection and characterization of resistance to pristinamycin in *Mycoplasma genitalium*

**DOI:** 10.1128/aac.00094-26

**Published:** 2026-03-31

**Authors:** Chloé Le Roy, Jennifer Guiraud, Otgonjargal Byambaa, Carla Balcon, Léo Gillet, Jorgen S. Jensen, Cécile Bébéar, Sabine Pereyre, On behalf of the European Society of Clinical Microbiology and Infectious Diseases (ESCMID) Study Group for Mycoplasma and Chlamydia Infections (ESGMAC)

**Affiliations:** 1 University of Bordeaux, CNRS, MFP, Fundamental Microbiology and Pathology, UMR 5234, Bordeaux, France; 2 Bacteriology Department, Bordeaux University Hospital, National Reference Centre for bacterial sexually transmitted infections 614403, Bordeaux, France; 3 Department of Microbiology and Infection prevention control, Mongolian National University of Medical Science, School of Biomedicine 106188, Ulaanbaatar, Mongolia; 4 Department of Bacteria, Parasites and Fungi, Statens Serum Institut 4326, Copenhagen, Denmark; University of Fribourg, Fribourg, Switzerland

**Keywords:** *Mycoplasma genitalium*, pristinamycin, josamycin, resistance, sexually transmitted infection

## Abstract

Pristinamycin, a streptogramin combination, is recommended as a third-line treatment for *Mycoplasma genitalium* infections according to European guidelines. However, the molecular mechanisms underlying pristinamycin resistance have not yet been elucidated. We investigated the *in vitro* development of pristinamycin-resistant *M. genitalium* mutants under exposure to subinhibitory concentrations of pristinamycin or josamycin. Resistant mutants were characterized by determining the MICs of seven antibiotics, performing Sanger sequencing of 23S rRNA and the ribosomal protein L4 and L22 genes, and conducting whole-genome sequencing in comparison with the parental, susceptible G37 reference strain. Mutants selected in the presence of pristinamycin harbored either an A2062C or A2062G mutation in 23S rRNA, both associated with a marked increase in the MICs of pristinamycin and josamycin. By contrast, selection with josamycin produced a mutant carrying the A2059G mutation, which exhibited elevated MICs for erythromycin, azithromycin, josamycin, and clindamycin, but not for pristinamycin. None of the three resistant mutants showed any detectable growth defect in culture. To mimic pristinamycin treatment in a macrolide-resistant background, further selection was performed using subinhibitory concentrations of pristinamycin on the azithromycin-resistant A2059G mutant. This led to the emergence of an additional C2611T substitution in 23S rRNA, resulting in a pristinamycin MIC of 8 mg/L. No stable mutations were observed in the ribosomal protein L4 or L22 genes during the selection process. In conclusion, this study demonstrates that pristinamycin resistance in *M. genitalium* is mediated by mutations in the 23S rRNA gene. These laboratory-derived mutations may foreshadow resistance mechanisms emerging in clinical isolates.

## INTRODUCTION

*Mycoplasma genitalium* is a recognized sexually transmitted organism associated with 15%–25% cases of non-gonococcal urethritis in men (1). In women, it is linked to cervicitis and pelvic inflammatory disease. Only a few antimicrobial classes are active against *M. genitalium*, including macrolides, fluoroquinolones, tetracyclines, and streptogramin combinations (2). Azithromycin, a macrolide, is the first-line treatment, while moxifloxacin is the recommended second-line option for macrolide-resistant *M. genitalium* infections (2, 3). However, resistance to these antibiotics has been increasing over recent decades (4). Acquired resistance to macrolides is associated with point mutations in domain V of the 23S rRNA gene (3), whereas mutations in the genes encoding ribosomal proteins L4 and L22 have, to date, an unconfirmed role in macrolide resistance in *M. genitalium* (5, 6). Moxifloxacin treatment failure has been linked to mutations in the *parC* gene, particularly the Ser83Ile substitution, which is sometimes accompanied by mutations in the *gyrA* gene (3). The rising prevalence of dual resistance in *M. genitalium* has led to its recognition as an emerging global health threat and its inclusion on the US CDC Antibiotic Resistance Threats Watch List in 2019.

Pristinamycin, a streptogramin combination, is recommended as third-line treatment for patients in whom azithromycin and moxifloxacin failed according to the French (https://www.has-sante.fr/jcms/p_3604652/fr/traitement-curatif-des-personnes-infectees-par-mycoplasma-genitalium) and European guidelines (2). Because of the difficulty in isolating and culturing *M. genitalium* from clinical specimens, very limited data on pristinamycin susceptibility have been published (7, 8). Notably, the current prevalence and geographical distribution of pristinamycin treatment failure remain largely unknown. Only four studies from Australia and the UK have reported microbiological cure rates of 66% to 75% with pristinamycin in patients infected with macrolide-resistant strains (7, 9–11). These findings underscore the need for a better understanding of the mechanisms underlying pristinamycin resistance. To date, however, no data are available on the mechanisms of pristinamycin resistance in *M. genitalium*. In *M. pneumoniae*, a phylogenetically related species, cross-resistance to pristinamycin and josamycin, a 16-membered macrolide has been associated with a mutation in the 23S rRNA gene at position 2062 (*Escherichia coli* numbering) (12).

To address this knowledge gap, we investigated the *in vitro* development of pristinamycin-resistant *M. genitalium* mutants under exposure to subinhibitory concentrations of pristinamycin or josamycin. The selected resistant mutants were characterized by determining the MICs of seven antibiotics, performing Sanger sequencing of the 23S rRNA and ribosomal protein L4 and L22 genes, and conducting whole-genome sequencing (WGS) for comparison with the parental strain sequences.

## MATERIALS AND METHODS

### Bacterial strains, growth conditions, and antibiotics

The *M. genitalium* reference strain G37 (ATCC 33530) was cultured at 37°C in 2 mL of FRIIS broth medium (Merck). The following antimicrobial agents were obtained from the indicated manufacturers: erythromycin (Sigma-Aldrich), azithromycin (Sigma-Aldrich), josamycin (Farmalyon), clindamycin (Sigma-Aldrich), pristinamycin (Aventis), doxycycline (Sigma-Aldrich), and moxifloxacin (Sigma-Aldrich).

### Selection of macrolide-resistant mutants from *M. genitalium* G37 and MIC determination

**Fig 1:**
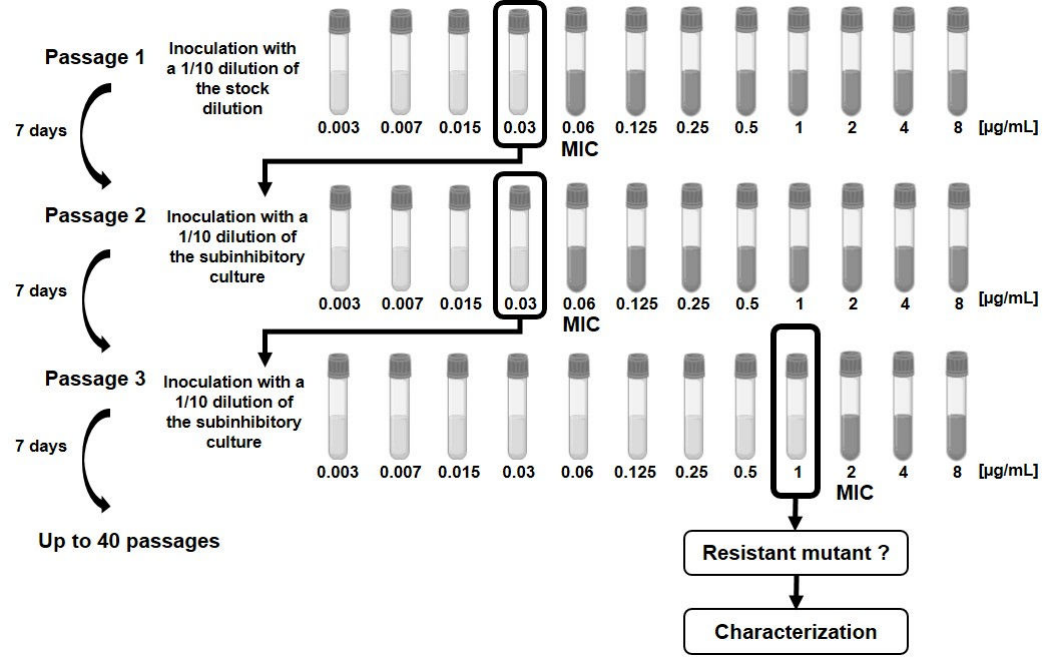
Experimental design of the *in vitro* selection process in axenic culture.

Resistant mutants were selected through serial passages of the parental *M. genitalium* G37 reference strain in FRIIS medium containing subinhibitory concentrations of josamycin or pristinamycin, as previously described (12). The MIC of the selecting antibiotic was determined after 7 days when the antibiotic-free growth control exhibited a color change (Fig. 1). The corresponding subinhibitory concentration was then used for the next passage. All culture tubes containing inhibitory concentrations were maintained at 37°C for up to 1 month, with growth monitored daily by observing color change. No subcultures were performed during the incubation period. When a color change occurred in a culture tube containing an antibiotic concentration at least fourfold higher than the MIC, molecular characterization was performed (see below). Once mutants had been obtained, 10 consecutive subcultures were carried out in antibiotic-free medium.

For each selected mutant and the G37 parental strain, the MICs of seven antibiotics (erythromycin, azithromycin, josamycin, clindamycin, pristinamycin, moxifloxacin, and doxycycline) were determined using the broth microdilution method as previously described (12). The MICs for all seven drugs were also determined after the 10 antibiotic-free subcultures to assess whether the phenotype remained stable in the absence of antibiotic selection pressure. The persistence of the selected mutations was confirmed by Sanger sequencing after these subcultures (see below).

To mimic pristinamycin treatment in a macrolide-resistant background, further selection was performed using subinhibitory concentrations of pristinamycin on the A2059G mutant (J10 mutant) obtained through josamycin selection. The J10 mutant underwent 40 serial passages in subinhibitory concentrations of pristinamycin, followed by 10 passages in antibiotic-free medium.

### Molecular characterization of resistant mutants

A fragment of the 23S rRNA gene encompassing positions 2058/2059, along with the complete coding sequences of the ribosomal protein L4 (*rplD*) and L22 (*rplV*) genes, was amplified as previously described (13). A second fragment of the 23S rRNA gene, encompassing position 2611, was amplified using primers MG/MPN-23S-F3 (5′GGATAAAAGCTACTCCGGG3′) and MG-23S-R4 (5′GATAAATACCTTTTATCAACC3′). All polymerase chain reaction (PCR) amplicons were analyzed by Sanger sequencing (Eurofins Genomics).

### WGS of resistant mutants

Genomic DNA was extracted from 5 mL cultures of the selected resistant mutants and the G37 parental strain using Genomic DNA and RNA Purification AXG20 columns and the Nucleobond Buffer Set III/IV (Macherey-Nagel). WGS was performed using short-read (Illumina) and long-read (Oxford Nanopore) technologies. Sequence libraries were prepared with the Illumina DNA Prep kit and sequenced on the Illumina iSeq 100 platform. Long-read sequencing was carried out by Eurofins Genomics using the GridION platform. Raw sequencing reads were mapped to the *M. genitalium* reference strain G37 genome assembly (ASM2732v1) using BWA (v0.7.17). Samtools (v1.19.2) was employed to index, sort, and mark duplicates in the alignment files. Bayesian variant detection was performed with FreeBayes (v1.3.6) and variant calling with BCFtools (v1.19), applying a minimum base depth of 10, a minimum mapping quality of 60, and a minimum base quality of 30. All resulting files were processed into readable reports using a set of in-house scripts, and consensus sequences were extracted. Sequence variations in the mutants including single-nucleotide polymorphisms (SNPs), insertions, and deletions were compared with those of the parental strain, and differences were recorded. High-quality genome assemblies and raw sequencing reads for the selected resistant mutants have been deposited in NCBI’s Genome and Sequence Read Archive under BioProject number PRJNA1104570.

### Growth of resistant mutants

To assess fitness, the selected mutants and the parental G37 reference strain were cultured for 14 days in 5 mL of antibiotic-free FRIIS medium. Each day, a 200 µL sample was collected and subjected to DNA extraction using the Nucleospin Tissue Kit (Macherey-Nagel). The *M. genitalium* load was quantified by an in-house TaqMan real-time PCR assay targeting the MgPa adhesin gene (14), following the construction of a calibrator plasmid named pMgPa. Briefly, a 78-bp fragment amplified from the MgPa gene of the *M. genitalium* G37 strain (14) was purified using the Wizard PCR Preps DNA Purification System (Promega) and subsequently cloned into the plasmid vector pGEM-T Easy (Promega) according to the manufacturer’s instructions. Ten-fold serial dilutions of pMgPa, ranging from 4 to 4 × 10^6^ copies/µL, were used to generate a standard curve. The concentration of *M. genitalium* DNA was then calculated as genome equivalents per microliter using the pMgPa standard curve on a LightCycler 480 real-time PCR instrument (Roche Diagnostics).

## RESULTS

### *In vitro* selection of resistant mutants from the wild-type G37 reference strain

Resistant mutants were selected through serial passages of the parental *M. genitalium* G37 strain in the presence of subinhibitory concentrations of josamycin or pristinamycin. The MICs of josamycin and pristinamycin for the parental *M. genitalium* G37 strain were 0.007 mg/L and 0.25 mg/L, respectively (Table 1).

**TABLE 1:** Characteristics of *in vitro*-selected resistant mutants of *M. genitalium*^*c*^

Strain designation	Selecting antibiotic, number of passages	Incubation time (days)	Antibiotic concentration of culture tube in which mutant was selected (mg/L)	MIC (mg/L)							Mutation		
				ERY	AZI	JOS	CLI	PRI	MOX	DOX	23S rRNA^*a*^	L4 protein (*rplD* gene)	L22 protein (*rplV* gene)
G37	Parental strain	–	–	0.007	≤ 0.003	0.007	0.25	0.25	0.125	0.125	WT	WT	WT
J10	JOS, 10	6	4	**32**	**32**	**32**	**16**	0.125	0.125	0.125	A2059G	WT	WT
P21A	PRI, 21	20	1	0.03	≤ 0.003	**16**	0.125	**2**	0.25	0.06	A2062G	WT	WT
P24	PRI, 24	18	2	**0.06**	≤ 0.003	**32**	0.5	**8**	0.125	0.25	A2062C	WT	WT
J10P40	JOS, 10 then PRI, 40^*b*^	7	2	**32**	**32**	**32**	**16**	**8**	0.125	0.125	A2059G C2611T	WT	WT

^*a*^ *Escherichia coli* numbering.

^*b*^ The parental strain for selection of the J10P40 mutant was the J10 mutant previously obtained after 10 passages with josamycin. The J10 mutant was subsequently exposed to 40 additional passages with subinhibitory concentrations of pristinamycin.

^*c*^ MICs showing an eight-fold or greater increase compared with the G37 parental strain are indicated in bold. AZI, azithromycin; CLI, clindamycin; DOX, doxycycline ERY, erythromycin; JOS, josamycin; MOX, moxifloxacin; PRI, pristinamycin; WT, wild-type; –, not applicable.

Using josamycin as the selecting antibiotic, mutant J10 was obtained at passage 10, showing a markedly increased josamycin MIC of 32 mg/L. Sanger sequencing identified an A2059G substitution in the 23S rRNA gene, while no alterations were detected in the L4 or L22 protein genes (Table 1).

For pristinamycin selection, the pristinamycin MIC remained stable, fluctuating between 0.125 and 0.25 mg/L over 30 passages. However, culture tubes containing inhibitory concentrations of pristinamycin were maintained at 37°C for up to 1 month and inspected daily for color changes. A color change was observed after 10–23 days of incubation in culture tubes containing pristinamycin concentrations ranging from 0.5 to 4 mg/L between passages 21 and 30. Molecular characterization of all cultures that grew in the presence of pristinamycin concentrations above 0.5 mg/L was performed between passages 21 and 26 (Table 2). This analysis revealed either wild-type 23S rRNA sequences, strains harboring an A2062G substitution, an A2062C substitution, or mixed populations containing combinations of wild-type, A2062G, and A2062C substitutions. Almost all isolates growing at pristinamycin concentrations ≥0.5 mg/L showed either the A2062G or the A2062C mutation (Table 2). Ultimately, at passage 21, after 20 days of incubation in the culture tube containing 1 mg/L pristinamycin, mutant P21A harboring a single A2062G substitution was selected (Table 2). Similarly, at passage 24, after 18 days of incubation in the culture tube containing 2 mg/L pristinamycin, mutant P24 carrying a single A2062C substitution was obtained. No mutations were detected in the L4 or L22 proteins of mutants P21A and P24.

**TABLE 2:** Polymorphism analysis of 23S rRNA, and L4 and L22 proteins using Sanger sequencing in cultures exhibiting a color change beyond 7 days during the *in vitro* selection of pristinamycin-resistant mutants from the G37 parental strain

No. of passage^*a*^	Pristinamycin concentration in culture tube (mg/L)	Incubation time (days)	23S rRNA^*b*^	L4 protein	L22 protein
21	0.125	10	WT	WT	WT
21	0.25	14	WT	WT	WT
21	0.5	18	WT/A2062G/C	WT	WT
**21 (P21A mutant**)	**1**	**20**	**A2062G**	**WT**	**WT**
21	2	23	A2062C	WT	WT
22	0.5	16	WT/A2062G/C	WT	WT
22	1	17	A2062C	WT	WT
22	2	21	A2062C	WT	WT
22	4	23	A2062C	WT	WT
23	0.5	14	A2062G	WT	WT
23	1	15	A2062G	WT	WT
23	2	20	A2062C	WT	WT
23	4	22	A2062C	WT	WT
24	0.5	12	A2062G/C	WT	WT
24	1	13	A2062G/C	WT	WT
**24 (P24 mutant**)	**2**	**18**	**A2062C**	**WT**	**WT**
24	4	22	A2062G/C	WT	WT
25	0.5	14	WT	WT	WT
25	1	18	A2062G/C	WT	WT
25	2	19	A2062C	WT	WT
25	4	21	A2062G/C	WT	WT
26	0.5	18	A2062G/C	WT	WT
26	1	19	A2062C	WT	WT
26	2	22	A2062C	WT	WT
26	4	22	WT/A2062C	WT	WT

^*a*^ Pristinamycin-resistant mutants selected for further phenotypic and genotypic characterization are shown in bold.

^*b*^ *E. coli* numbering*.*

### Characterization of resistant mutants

The MICs for seven antibiotics were determined for all selected mutants (Table 1). The J10 mutant displayed a marked increase in the MICs of erythromycin, azithromycin, josamycin, and clindamycin (a ≥64-fold rise for all 4 antibiotics), while the pristinamycin MIC remained unchanged at 0.125 mg/L. Both P21A and P24 mutants showed a substantial increase in pristinamycin MICs (8- and 32-fold increases, respectively) and in josamycin MICs (16 mg/L and 32 mg/L, respectively, compared with 0.007 mg/L for the parental strain). A moderate four- to eight-fold rise in erythromycin MICs was also observed. By contrast, azithromycin, clindamycin, doxycycline, and moxifloxacin MICs remained unchanged for both mutants.

Resistance phenotypes and associated mutations remained stable in all three mutants after 10 consecutive subcultures in antibiotic-free medium.

**Fig 2:**
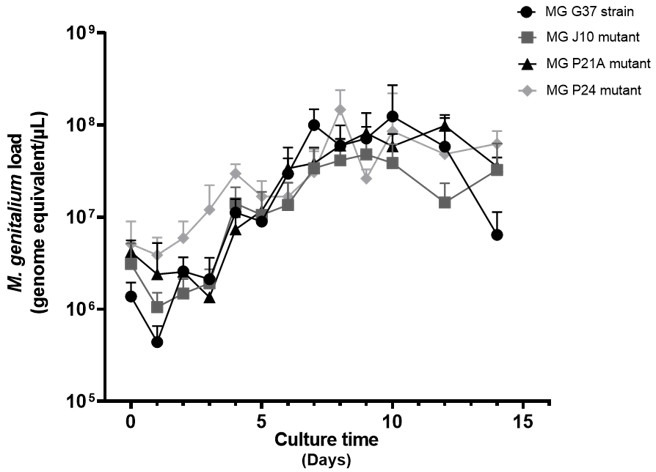
Growth curves of *M. genitalium* G37 reference strain and resistant mutants. Strains were cultured in antibiotic-free FRIIS medium. *M. genitalium* load was quantified using MgPa real-time quantitative PCR [14]. Data represent mean ± standard deviation from three independent cultures.

Comparison of the growth curves of the J10, P21A, and P24 mutants with that of the parental G37 strain revealed no detectable growth defect in axenic culture (Fig. 2; Table S1).

### Whole-genome analysis of resistant mutants

The whole genomes of the J10, P21A, and P24 mutants were compared with that of the parental G37 strain to determine whether additional changes beyond the 23S rRNA mutations could account for the resistance phenotype. WGS confirmed the presence of 23S rRNA mutations associated with macrolide resistance. Mutant J10 harbored an A2059G substitution, present in 100% of reads; P21A carried an A2062G mutation in 100% of reads; and P24 exhibited a mixed population, with A2062C detected in 93% and A2062G in 7% of reads. No additional mutations were identified in the 23S rRNA gene, and the absence of mutations in the *rplD* and *rplV* genes was confirmed for all three mutants. Beyond the macrolide resistance-associated 23S rRNA mutations, seven, three, and seven additional sequence variations were identified in comparison with our laboratory G37 strain in mutants J10, P21A, and P24, respectively. These variations were distributed across 10 coding DNA sequences and two intergenic regions (Table 3).

**TABLE 3:** Genetic alterations detected using whole-genome analysis in the *M. genitalium*-resistant J10, P21A, and P24 mutants selected from the G37 parental strain^*a*^^,^^*d*^

Location in G37 genome^*b*^	Locus tag (gene name)	Protein product	Nucleotide // amino acid alteration		
			J10	P21A	P24
16,357	MG_RS00080	ABC transporter ATP-binding protein	–	–	C803T // Pro268Leu
16,377	MG_RS00080	ABC transporter ATP-binding protein	A823G // Ser275Gly	–	–
116,650	MG_RS00495 (*fusA*)	Elongation factor G	WT	G1843A // Asp615Asn	G1843A // Asp615Asn
173,799	MG_RS00780	**23S ribosomal RNA**	**A2072G^*c*^**	–	–
173,802	MG_RS00780	**23S ribosomal RNA**	–	**A2075G^*c*^**	**A2075C^*c*^**
197,546	MG_RS00935 (*rpsH*)	30S ribosomal protein S8	G370A // Asp124Asn	–	–
263,187	MG_RS01280 (*hmw2*)	Terminal organelle tip protein HMW2	–	–	C4011T // Tyr1337Tyr
270,420	MG_RS01325	APC family permease	C16T // Arg6Trp	–	–
349,735	Intergenic region	Non-coding region	TTTC insertion	–	–
350,815	MG_RS01730	MgpC family cytadherence protein	–	–	C391G // Pro131Ala
351,007	MG_RS01730	MgpC family cytadherence protein	G583T // Asp195Tyr	–	–
446,624	MG_RS02140	Replication initiation and membrane attachment family protein	–	–	C7G // Pro3Ala
447,068	MG_RS02140	Replication initiation and membrane attachment family protein	–	A451G // Asn151Asp	–
493,769	MG_RS02380	Leucyl aminopeptidase	–	G1199T // Arg400Ile	G1199T // Arg400Ile
496,991	Intergenic region	Non-coding region	TTTAAAAG insertion	–	–
530,583	MG_RS02530	RNase J family beta-CASP ribonuclease	C635T // Ser212Leu	–	–
548,410	MG_RS02620	YitT family protein	–	–	C442T // Pro148Ser

^*a*^ Mutations in bold were previously found using Sanger sequencing.

^*b*^ The genome of the parental *M. genitalium* G37 strain maintained in our laboratory was first compared with the GenBank reference sequence. Only two genetic variations were identified: (i) at genome position 156,617, a T52C substitution in the *rny* gene (MG_RS0075, encoding ribonuclease Y), resulting in an F18L amino acid change and (ii) at genome position 432,007, a T2452G substitution in the *rpoC *gene (MG_RS02085, encoding DNA-directed RNA polymerase subunit β), leading to a C818G amino acid change. These two SNPs are present in all mutants and are not listed in the table.

^*c*^ 23S rRNA mutations at positions 2072 and 2075 in *M. genitalium* numbering correspond to positions 2059 and 2062 in *E. coli* numbering, respectively.

^*d*^ –, no alteration (wild-type sequence).

In both pristinamycin-resistant mutants, P21A and P24, a single G-to-A base change was detected at position 1843 of the *fusA* gene, which encodes FusA (also known as elongation factor G), predicting a D615N substitution (Table 3). The fusidic acid MIC was identical for the G37, P21A, and P24 strains (2 mg/L). Consistent with this observation, the UniProt database (https://www.uniprot.org/) did not indicate any impact of the D615N mutation on protein activity.

### *In vitro* selection of pristinamycin-resistant mutant from the macrolide-resistant J10 mutant

To evaluate the potential for a macrolide-resistant mutant to acquire additional resistance to pristinamycin upon drug exposure, the macrolide-resistant J10 mutant was subjected to 40 serial passages in subinhibitory concentrations of pristinamycin. This *in vitro* procedure was designed to simulate current therapeutic guidelines for infections caused by *M. genitalium* strains resistant to macrolides. After each passage, cultures grown at inhibitory pristinamycin concentrations were maintained at 37°C for up to 30 days. The MIC of pristinamycin initially ranged between 0.125 and 0.5 mg/L over the first 13 passages and then progressively increased to values between 1 and 8 mg/L. However, no additional mutations were detected in the 23S rRNA fragment encompassing positions 2058/2059, nor in the *rplD* or *rplV* genes. From passages 17 to 35, double peaks were observed on chromatograms of the *rplV* (L22) gene, indicating mixed populations carrying wild-type sequences alongside amino acid substitutions S53R, S81R, A87V, or S108F (*M. genitalium* numbering) (Table S2). These mutations were no longer detected in protein L22 after passage 35.

Selection was halted after passage 40, and the resulting J10P40 strain obtained after 7 days of incubation in 2 mg/L pristinamycin was fully characterized. Compared with the parental J10 mutant, the J10P40 strain displayed identical MICs for erythromycin, azithromycin, josamycin, and clindamycin (16–32 mg/L for all four antibiotics) (Table 1). However, the J10P40 mutant exhibited a marked 64-fold increase in pristinamycin MIC (8 mg/L), while doxycycline and moxifloxacin MICs remained unchanged. These MIC values remained stable after 10 additional passages in antibiotic-free medium. WGS revealed three mutations in the 23S rRNA gene: the A2059G substitution already present in the J10 mutant, and two new mutations C2611T in domain V and C1782T in domain IV (*E. coli* numbering). Comparative genomic analysis between the J10 and J10P40 mutants identified several additional SNPs and indels, primarily located in variable regions such as adhesin genes, which were unlikely to contribute to pristinamycin resistance (Table S3). Given the potential role of the C2611T substitution in pristinamycin resistance, retrospective Sanger sequencing of the 23S rRNA region encompassing position 2611 was performed across all 40 passages (Table S2). The C2611T mutation first appeared at passage 16. Between passages 16 and 24, this mutation emerged sporadically and was detected only in cultures exposed to pristinamycin concentrations of ≥2 mg/L, corresponding to a 10-fold increase over the initial MIC. From passages 25 to 29, mixed populations containing both wild-type and C2611T mutant sequences were observed. After passage 30, the C2611T mutation became fixed in the population, with consistent growth observed in 8 mg/L of pristinamycin.

## DISCUSSION

Culturing *M. genitalium* from clinical specimens is challenging because of low success rates and long turnaround times, which limits the use of phenotypic antimicrobial susceptibility testing to a few specialized laboratories (15). Consequently, resistance is routinely inferred using molecular assays in clinical practice (1, 2, 16) although culture remains an essential tool for research. In this study, the axenically cultivable G37 reference strain was used for *in vitro* selection of resistant mutants. This approach enables the investigation of resistance mechanisms, particularly for antibiotics such as pristinamycin, for which the underlying mechanisms remain poorly defined and access to clinical isolates from treatment failures is limited. Moreover, the use of an isogenic background allows resistance to be studied without the added complexity and genetic variability typical of clinical isolates.

In this study, josamycin was used as the selecting antibiotic with the aim of inducing pristinamycin resistance. This choice was based on previous *in vitro* selection studies employing subinhibitory concentrations of josamycin in *M. hominis* and *M. pneumoniae*, which identified A2062G and A2062T mutations, associated with high-level resistance to both josamycin and pristinamycin (12, 17). However, in *M. genitalium*, no mutations at position 2062 were selected in the presence of josamycin. Because only a single selection experiment was performed with josamycin in this study, we cannot rule out the possibility that additional selection assays might have produced resistant mutants carrying substitutions at position 2062. Nevertheless, our findings demonstrate that josamycin can select for the A2059G substitution in *M. genitalium* relatively early in the selection process. The A2059G substitution, together with A2058G, represents the most common 23S rRNA mutations identified in *M. genitalium*, conferring resistance to the 15-membered macrolide azithromycin (3, 5), with azithromycin MICs of ≥8 mg/L in clinical isolates (18, 19). The A2059G mutation has also been shown to confer high-level resistance to 16-membered macrolides such as spiramycin, midecamycin, and tylosin in the closely related species *M. pneumonia* (20). In the present study, the A2059G mutant exhibited high-level resistance to 14-, 15-, and 16-membered macrolides, with MIC values of 32 mg/L, as well as resistance to clindamycin, but no increase in pristinamycin MIC.

Using subinhibitory concentrations of pristinamycin, we successfully selected pristinamycin-resistant *M. genitalium* mutants. To date, no clinical breakpoints or epidemiological cutoff values (ECOFFs) have been established for pristinamycin against *M. genitalium* by either CLSI or EUCAST. By analogy with *Staphylococcus*, *Enterococcus*, and *Streptococcus* species, for which the clinical breakpoint is set at 1 mg/L according to the 2025 recommendations of the CA-SFM (21), MICs >1 mg/L should raise clinical concern and be interpreted as indicating decreased susceptibility, with a likely risk of treatment failure under standard oral regimens. We demonstrated for the first time that the A2062G and A2062C substitutions in the 23S rRNA gene are associated with pristinamycin resistance in *M. genitalium*, consistent with the drug’s binding at position A2062 within the peptidyl transferase loop of 23S rRNA (22). The pristinamycin-resistant mutants were also resistant to josamycin but remained susceptible to 15-membered macrolides. A slight increase in the MICs of 14-membered macrolides was observed, reflecting minor differences in binding sites among macrolide subclasses (23). Although the mutants characterized in this study were generated under laboratory conditions, the A2062G (24) and A2062T (25) substitutions have already been detected in *M. genitalium*-positive clinical specimens. Moreover, an association between the A2062T substitution and pristinamycin treatment failure has been reported (26). In clinical practice, pristinamycin is usually used as a last-line therapy for patients who have previously been exposed to multiple macrolide and fluoroquinolone regimens. Such treatment histories may favor the acquisition of additional 23S rRNA mutations under sustained antibiotic pressure.

WGS was employed to investigate comprehensively the mechanisms of antibiotic resistance in the *in vitro*-selected mutants. Genome analysis revealed several additional SNPs located in various protein-coding genes and intergenic regions. To our knowledge, these substitutions are unlikely to be associated with resistance to macrolides or related antibiotics. The distinct genetic variations observed between josamycin-resistant and pristinamycin-resistant mutants suggest that the parental G37 strain followed different evolutionary trajectories under distinct selective pressures. Furthermore, a mutation in the *fusA* gene was identified in both pristinamycin-resistant mutants, P21A and P24. Fusidic acid inhibits bacterial growth by binding to elongation factor G (encoded by fusA), thereby blocking protein synthesis (27). A recent study reported that certain *fusA* mutations are associated with fusidic acid resistance in *M. genitalium* (28). In the present study, the single-nucleotide mutation identified in *fusA* was not associated with an increase in the fusidic acid MIC. This finding is consistent with the mutation being located outside the fusidic acid binding site (27). Our results also align with the findings of Wood et al. (28), who indicated that macrolide resistance does not influence fusidic acid susceptibility.

Following the selection process, serial passages of the mutants in antibiotic-free medium demonstrated the *in vitro* stability of all resistance-associated mutations in the 23S rRNA gene, along with their corresponding MICs. Genetic alterations are often associated with a fitness burden referred to as the biological cost that can impair bacterial growth capacity (29). In the absence of antibiotic selection pressure, susceptible bacteria might therefore be expected to outcompete resistant mutants; however, this was not observed in the present study. Furthermore, comparison of the growth kinetics between the susceptible parental G37 strain and its isogenic resistant mutants revealed no detectable growth defects, suggesting that these 23S rRNA mutations do not impose a measurable biological cost under laboratory conditions.

Finally, to mimic pristinamycin treatment in a macrolide-resistant background, further selection was carried out using subinhibitory concentrations of pristinamycin on the azithromycin-resistant A2059G mutant (mutant J10). After 40 passages, mutant J10 exhibited a pristinamycin MIC of 8 mg/L, associated with two additional mutations in the 23S rRNA gene: C1782T and C2611T. The C1782T substitution is located outside the macrolide-binding sites in 23S rRNA (23) and, to our knowledge, has never been associated with resistance to macrolides or related antibiotics in any bacterial species. By contrast, the cytosine at position 2611 of 23S rRNA forms a base pair with residue 2057 and contributes to the local binding conformation for macrolides (30). The C2611T substitution has previously been reported in *in vitro*-selected macrolide-resistant mutants of *M. hominis* (30), where its association with A2059G was observed but did not affect the pristinamycin MIC. In *M. agalactiae*, the same C2611T and A2059G combination was shown to increase MICs for macrolides and lincosamides (31). Notably, the C2611T substitution has already been identified in *M. genitalium*-positive clinical specimens and was presumed to contribute to macrolide resistance although direct evidence has been lacking (32). In our study, the fixation of the C2611T substitution after passage 30, together with the consistent growth of mutants in 8 mg/L pristinamycin, supports its role in increasing the pristinamycin MIC. Nevertheless, to confirm the involvement of the C2611T substitution in pristinamycin resistance, it would be valuable to investigate its presence in clinical specimens from the approximately 25% of patients who experience pristinamycin treatment failure (7) and, where possible, perform antimicrobial susceptibility testing on successfully cultured isolates.

In mutant J10P40, four distinct point mutations were identified in the L22 gene, including an S81R substitution (Table S2). In the literature, only an S81T substitution has previously been reported, in a patient with *M genitalium* urethritis, but it was not associated with macrolide resistance (6). Overall, to date, L22 mutations reported in *M. genitalium* have not been conclusively linked to resistance to macrolides or related antibiotics (5, 6). Moreover, the instability of L22 mutations observed throughout the selection of pristinamycin-resistant mutants (Table S2) suggests that these alterations are unlikely to contribute to the increased pristinamycin MIC. Notably, L22 mutations were no longer present in the J10P40 mutant.

Overall, this study demonstrates that pristinamycin resistance in *M. genitalium* is mediated by mutations in the 23S rRNA gene. Specifically, single substitutions at position 2062, as well as the combination of C2611T with A2059G, were associated with a marked increase in pristinamycin MICs. These findings raise concerns that currently available commercial assays, which detect only a limited set of four to six macrolide resistance-associated mutations located at positions A2058 and A2059 (16, 33, 34), may fail to identify emerging pristinamycin-resistant strains. Incorporating the detection of mutations at positions A2062 and C2611 of the 23S rRNA gene into existing real-time PCR or hybridization-based commercial assays would improve surveillance of pristinamycin resistance and support appropriate antimicrobial selection for patients infected with macrolide- and moxifloxacin-resistant strains.

In conclusion, the laboratory-derived mutants generated in this study may foreshadow mutations that could emerge in clinical strains and potentially contribute to pristinamycin treatment failure. Because *in vitro* models may not fully reflect the complexity of clinical infections, further investigations using patient-derived isolates are needed to refine these findings.

## Data Availability

Data used in this study are available upon request from the corresponding authors.

## SUPPLEMENTAL MATERIAL

Supplemental tables aac.00094-26-s0001.docx Tables S1 to S3.

ASM does not own the copyrights to Supplemental Material that may be linked to, or accessed through, an article. The authors have granted ASM a non-exclusive, world-wide license to publish the Supplemental Material files. Please contact the corresponding author directly for reuse.
