# Supplementary material for: In vitro selection and characterization of resistance to pristinamycin in Mycoplasma genitalium
Source: Antimicrob Agents Chemother. 2026 Mar 31;70(5):e00094-26. doi: 10.1128/aac.00094-26 (PMC13148045; doi:10.1128/aac.00094-26)
Supplement: Supplemental tables — Tables S1 to S3. [file aac.00094-26-s0001.docx]

**Supplementary materials**

**TABLE S1. Load of *M. genitalium* G37 reference strain and resistant mutants during 14 days of incubation.**

Cultures were grown in triplicate in antibiotic-free FRIIS medium. *Mycoplasma genitalium* load was quantified using a quantitative real-time PCR assay targeting the MgPa adhesin gene.^13^

| **Growth time (days)** | **G37 load**  **(genome equivalents/µL)** | | **J10 load**  **(genome equivalents/µL)** | | **P21A load**  **(genome equivalents/µL)** | | **P24 load**  **(genome equivalents/µL)** | |
| --- | --- | --- | --- | --- | --- | --- | --- | --- |
|  | Mean | SD | Mean | SD | Mean | SD | Mean | SD |
| 0 | 1.38 × 10^6^ | 5.71 × 10^5^ | 3.11 × 10^6^ | 2.09 × 10^6^ | 4.17 × 10^6^ | 1.44 × 10^6^ | 5.12 × 10^6^ | 3.88 × 10^6^ |
| 1 | 4.41 × 10^5^ | 2.14 × 10^5^ | 1.06 × 10^6^ | 4.57 × 10^5^ | 2.40 × 10^6^ | 2.84 × 10^6^ | 3.88 × 10^6^ | 2.13 × 10^6^ |
| 2 | 2.58 × 10^6^ | 1.09 × 10^6^ | 1.49 × 10^6^ | 6.52 × 10^5^ | 2.55 × 10^6^ | 9.64. × 10^4^ | 5.93 × 10^6^ | 3.07 × 10^6^ |
| 3 | 2.12 × 10^6^ | 1.50 × 10^6^ | 1.91 × 10^6^ | 8.02 × 10^5^ | 1.35 × 10^6^ | 1.39 × 10^5^ | 1.20 × 10^7^ | 1.02 × 10^7^ |
| 4 | 1.13 × 10^7^ | 4.45 × 10^6^ | 1.41 × 10^7^ | 7.08 × 10^6^ | 7.37 × 10^6^ | 2.63 × 10^6^ | 2.98 × 10^7^ | 7.82 × 10^6^ |
| 5 | 8.97 × 10^6^ | 1.01 × 10^6^ | 1.06 × 10^7^ | 8.23 × 10^6^ | 1.17 × 10^7^ | 7.23 × 10^5^ | 1.68 × 10^7^ | 7.85 × 10^6^ |
| 6 | 2.96 × 10^7^ | 2.74 × 10^7^ | 1.36 × 10^7^ | 1.01 × 10^7^ | 3.38 × 10^7^ | 9.66 × 10^6^ | 1.67 × 10^7^ | 6.77 × 10^6^ |
| 7 | 1.00 × 10^8^ | 4.86 × 10^7^ | 3.40 × 10^7^ | 2.15 × 10^7^ | 3.86 × 10^7^ | 1.83 × 10^7^ | 3.07 × 10^7^ | 2.13 × 10^7^ |
| 8 | 5.97 × 10^7^ | 1.12 × 10^7^ | 4.14 × 10^7^ | 2.85 × 10^7^ | 6.00 × 10^7^ | 3.94 × 10^7^ | 1.47 × 10^8^ | 9.26 × 10^7^ |
| 9 | 7.16 × 10^7^ | 6.37 × 10^7^ | 4.80 × 10^7^ | 2.63 × 10^7^ | 8.19 × 10^7^ | 1.37 × 10^7^ | 2.62 × 10^7^ | 6.84 × 10^6^ |
| 10 | 1.24 × 10^8^ | 1.47 × 10^8^ | 3.86 × 10^7^ | 2.33 × 10^7^ | 5.89 × 10^7^ | 2.13 × 10^7^ | 8.57 × 10^7^ | 1.35 × 10^8^ |
| 12 | 5.82 × 10^7^ | 6.10 × 10^7^ | 1.44 × 10^7^ | 8.96 × 10^6^ | 9.84 × 10^7^ | 3.02 × 10^7^ | 4.83 × 10^7^ | 4.36 × 10^7^ |
| 14 | 6.43 × 10^6^ | 4.92 × 10^6^ | 3.25 × 10^7^ | 3.04 × 10^7^ | 3.54 × 10^7^ | 9.08 × 10^6^ | 6.27 × 10^7^ | 2.33 × 10^7^ |

SD, standard deviation.

**TABLE S2. Polymorphism analysis of 23S rRNA, and L4 and L22 proteins using Sanger sequencing in cultures exhibiting a colour change at 7 days or later during the *in vitro* selection of pristinamycin-resistant mutants from the J10 mutant strain.**

| **No. of passage*** | **Pristinamycin concentration in culture tube (mg/L)** | **Incubation time (days)** | **23S rRNA fragment encompassing nt 2058/2059**** | **23S rRNA fragment encompassing nt 2611**** | **L4 protein***** | **L22 protein***** |  |
| --- | --- | --- | --- | --- | --- | --- | --- |
| 0 | 0.06 | 7 | A2059G | WT | WT | WT |  |
| 1 | 0.06 | 7 | A2059G | WT | WT | WT |  |
| 1 | 0.25 | 14 | A2059G | WT | WT | WT |  |
| 2 | 0.125 | 7 | A2059G | WT | WT | WT |  |
| 6 | 0.125 | 7 | A2059G | WT | WT | WT |  |
| 6 | 1 | 25 | A2059G | WT | WT | WT |  |
| 12 | 0.25 | 7 | A2059G | WT | WT | WT |  |
| 12 | 1 | 11 | A2059G | WT | WT | WT |  |
| 12 | 2 | 18 | A2059G | WT | WT | WT |  |
| 13 | 0.5 | 7 | A2059G | WT | WT | WT |  |
| 13 | 2 | 15 | A2059G | WT | WT | WT |  |
| 14 | 1 | 7 | A2059G | WT | WT | WT |  |
| 14 | 4 | 11 | A2059G | WT | WT | WT |  |
| 15 | 2 | 7 | A2059G | WT | WT | WT |  |
| 16 | 2 | 7 | A2059G | WT | WT | WT |  |
| 16 | 8 | 27 | A2059G | C2611T | WT | WT |  |
| 17 | 4 | 22 | A2059G | C2611T | WT | WT/S108F |  |
| 18 | 1 | 7 | A2059G | WT | WT | WT |  |
| 18 | 4 | 16 | A2059G | C2611T | WT | WT/S108F |  |
| 18 | 8 | 30 | A2059G | C2611T | WT | WT/S108F |  |
| 19 | 1 | 7 | A2059G | WT | WT | WT/S108F |  |
| 20 | 0.5 | 7 | A2059G | WT | WT | WT/A87V |  |
| 20 | 1 | 10 | A2059G | WT | WT | WT/S108F |  |
| 21 | 0.5 | 7 | A2059G | WT | WT | WT/S108F |  |
| 21 | 1 | 9 | A2059G | WT | WT | WT/S108F |  |
| 21 | 2 | 13 | A2059G | WT | WT | WT/S108F |  |
| 21 | 4 | 20 | A2059G | C2611T / WT | WT | WT/S108F |  |
| 22 | 2 | 7 | A2059G | WT | WT | WT/S108F |  |
| 22 | 4 | 10 | A2059G | C2611T / WT | WT | WT/S108F |  |
| 22 | 8 | 14 | A2059G | C2611T / WT | WT | WT/S108F |  |
| 23 | 1 | 7 | A2059G | WT | WT | WT/S108F |  |
| 23 | 2 | 10 | A2059G | WT | WT | WT/S108F |  |
| 23 | 4 | 15 | A2059G | C2611T / WT | WT | WT/S108F |  |
| 24 | 2 | 7 | A2059G | WT | WT | WT/S108F |  |
| 24 | 4 | 13 | A2059G | C2611T / WT | WT | WT/S108F |  |
| 24 | 8 | 20 | A2059G | C2611T | WT | WT/S108F |  |
| 25 | 4 | 7 | A2059G | C2611T / WT | WT | WT/S108F |  |
| 25 | 8 | 10 | A2059G | C2611T / WT | WT | WT/S108F |  |
| 26 | 2 | 7 | A2059G | C2611T / WT | WT | WT/S108F |  |
| 26 | 4 | 12 | A2059G | C2611T / WT | WT | WT/S108F |  |
| 26 | 8 | 12 | A2059G | C2611T / WT | WT | WT/S108F |  |
| 27 | 4 | 7 | A2059G | C2611T / WT | WT | WT |  |
| 27 | 8 | 13 | A2059G | C2611T / WT | WT | WT/S108F |  |
| 28 | 2 | 7 | A2059G | C2611T / WT | WT | WT/S108F |  |
| 28 | 4 | 10 | A2059G | C2611T / WT | WT | WT/S53R |  |
| 28 | 8 | 15 | A2059G | C2611T / WT | WT | WT/S108F |  |
| 29 | 8 | 7 | A2059G | C2611T / WT | WT | WT | |
| 30 | 2 | 7 | A2059G | C2611T | WT | WT/S108F | |
| 30 | 4 | 12 | A2059G | C2611T | WT | WT/S53R | |
| 31 | 2 | 7 | A2059G | C2611T | WT | WT/S53R | |
| 31 | 4 | 9 | A2059G | C2611T | WT | WT | |
| 31 | 8 | 30 | A2059G | C2611T | WT | WT/S53R | |
| 32 | 2 | 7 | A2059G | C2611T | WT | WT/S53R | |
| 32 | 4 | 14 | A2059G | C2611T | WT | WT/S53R | |
| 33 | 4 | 7 | A2059G | C2611T | WT | WT/S53R | |
| 33 | 8 | 12 | A2059G | C2611T | WT | WT/S81R | |
| 34 | 2 | 7 | A2059G | C2611T | WT | WT/S53R | |
| 34 | 4 | 10 | A2059G | C2611T | WT | WT | |
| 35 | 4 | 7 | A2059G | C2611T | WT | WT | |
| 35 | 8 | 14 | A2059G | C2611T | WT | WT/S53R | |
| 36 | 2 | 7 | A2059G | C2611T | WT | WT | |
| 36 | 4 | 12 | A2059G | C2611T | WT | WT | |
| 37 | 2 | 7 | A2059G | C2611T | WT | WT | |
| 37 | 4 | 9 | A2059G | C2611T | WT | WT | |
| 37 | 8 | 12 | A2059G | C2611T | WT | WT | |
| 38 | 1 | 7 | A2059G | C2611T | WT | WT | |
| 38 | 2 | 12 | A2059G | C2611T | WT | WT | |
| 38 | 4 | 28 | A2059G | C2611T | WT | WT | |
| 39 | 4 | 7 | A2059G | C2611T | WT | WT | |
| 39 | 8 | 14 | A2059G | C2611T | WT | WT | |
| **40**  **(J10P40 mutant)** | **2** | **7** | **A2059G** | **C2611T** | **WT** | **WT** | |
| 40 | 4 | 10 | A2059G | C2611T | WT | WT | |

*The pristinamycin-resistant mutant selected for further phenotypic and genotypic characterisation is shown in bold.

*******E. coli* numbering. ****M. genitalium* numbering.

Nt, nucleotide.

**TABLE S3. Comparison of genetic alterations between J10 and J10P40 resistant mutants based on whole-genome analysis.**

| **Location in G37 genome*** | **Locus tag (gene name)** | **Protein product** | **J10 mutant Nucleotide // Amino acid alteration** | **J10P40 mutant Nucleotide // Amino acid alteration** |
| --- | --- | --- | --- | --- |
| 16377 | MG_RS00080 | ABC transporter ATP-binding protein | A823G // Ser275Gly | A823G // Ser275Gly |
| 173518 |  |  | WT | C1791T |
| **173799** | **MG_RS00780 (rRNA)** | **23S ribosomal RNA** | **A2072G**** | **A2072G**** |
| **174351** |  |  | **WT** | **C2625T**** |
| 197546 | MG_RS00935 (*rpsH*) | 30S ribosomal protein S8 | G370A // Asp124Asn | G370A // Asp124Asn |
| 214970 |  |  | WT | 143_145delGCGinsCGA // SerVal48ThrMet |
| 214996 |  |  | WT | C169G // Leu57Val |
| 215001 |  |  | WT | A174G // Pro58Pro |
| 215007 |  |  | WT | 180_189delTACCAATGAGinsAAGTAGTGAC // ThrAsnGlu61SerSerAsp |
| 215028 | MG_RS02855 | Hypothetical protein | WT | 201_219delGTTGCAAGAATTGATCCTAinsACTGAAAAATTTAATTCTT // GlnGlu69LysAsn |
| 215055 |  |  | WT | 228_234delCCTTACCinsTTTGACT // 79 Synonymous |
| 215088 |  |  | WT | 261_267delTAAACAAinsCAAAGAG // Gln89Glu |
| 215100 |  |  | WT | T273C // Gly91Gly |
| 215106 |  |  | WT | C279G // Thr93Thr |
| 215118 |  |  | WT | G291A // Val97Val |
| 224006 |  |  | WT | 2437_2440delAAAGinsCAGC // LysGlu813GlnGln |
| 224068 | MG_RS01075 (*mgpB*) | Adhesin P140 | WT | 2499_2514delGTATCGCGTTCAAAGTinsTTATAGAAAA // Val836_Ser838delinsLys |
| 224090 |  |  | WT | 2521_2526delCAGAAAinsAACAACAAT // Gln841_Lys842delinsAsnAsnAsn |
| 226279 |  |  | WT | 365_367delCGAinsGCG // ThrMet122SerVal |
| 226305 |  |  | WT | G391C // Val131Leu |
| 226310 |  |  | WT | G396A // Pro132Pro |
| 226316 |  |  | WT | 402_411delAAGTAGTGACinsTACCAATGAG // SerSerAsp135ThrAsnGlu |
| 226337 |  |  | WT | 423_441delACTGAAAAATTTAATTCTTinsGTTGCAAGAATTGATCCTA // LysAsn143GlnGlu |
| 226364 | MG_RS01080 (*mgpC*) | Adhesin P110 | WT | 450_456delTTTGACTinsCCTTACC // synonymous |
| 226397 |  |  | WT | 483_489delCAAAGAGinsTAAACAA // Glu163Gln |
| 226409 |  |  | WT | C495T // Gly165Gly |
| 226415 |  |  | WT | G501C // Thr167Thr |
| 226427 |  |  | WT | A513G // Val171Val |
| 270420 | MG_RS01325 | APC family permease | C16T // Arg6Trp | C16T // Arg6Trp |
| 312702 | MG_RS01520 | P68 family surface protein | WT | G2192T // Arg731Leu |
| 349735 | Intergenic region | Non-coding region | T349735TTTC | T349735TTTC |
| 351007 | MG_RS01730 | MgpC family cytadherence protein | G583T // Asp195Tyr | G583T // Asp195Tyr |
| 496991 | Intergenic region | Non-coding region | T496991TTTAAAAG | T496991TTTAAAAG |
| 530583 | MG_RS02530 | RNase J family beta-CASP ribonuclease | C635T // Ser212Leu | C635T // Ser212Leu |

Mutations in bold were also found using Sanger sequencing.

*The genome of the parental *M. genitalium* G37 strain maintained in our laboratory was first compared with the GenBank reference sequence. Only two genetic variations were identified: (i) at genome position 156,617, a T52C substitution in the *rny* gene (MG_RS0075, encoding ribonuclease Y), resulting in an F18L amino acid change; and (ii) at genome position 432,007, a T2452G substitution in the *rpoC* gene (MG_RS02085, encoding DNA-directed RNA polymerase subunit β), leading to a C818G amino acid change. These two SNPs are present in all mutants and are therefore not included in the table.

**23S rRNA mutations at positions 2072 and 2625 in *M. genitalium* numbering correspond to positions 2059 and 2611 in *E. coli* numbering, respectively.

Del, deletion; ins, insertion; SNP, single-nucleotide polymorphism; WT, wild-type sequence.
